# Comparing the effect of sucrose gel and metronidazole gel in treatment of clinical symptoms of bacterial vaginosis: a randomized controlled trial

**DOI:** 10.1186/s13063-018-2905-z

**Published:** 2018-10-26

**Authors:** Somayyeh Khazaeian, Ali Navidian, Shahin-dokht Navabi-Rigi, Marzieh Araban, Faraz Mojab, Safoura Khazaeian

**Affiliations:** 1 Pregnancy Health Research Center, Department of Midwifery, Zahedan University of Medical Sciences, Zahedan, Iran; 2 Pregnancy Health Research Center, Department of Counseling, Zahedan University of Medical Sciences, Zahedan, Iran; 3 Social Determinants of Health Research Center, Ahvaz Jundishapur University of Medical Sciences, Ahvaz, Iran; 4 Department of Health Education and Promotion, Public Health School, Ahvaz Jundishapur University of Medical Sciences, Ahvaz, Iran; 5 Department of Pharmacognosy, School of Pharmacy, Shahid Beheshti University of Medical Sciences and Health Services, Tehran, Iran; 6 Department of Obstetrics and Gynecology, School of Medicine, Zahedan University of Medical Sciences, Zahedan, Iran

**Keywords:** Sucrose vaginal gel, Metronidazole vaginal gel, Bacterial vaginosis

## Abstract

**Background:**

*Lactobacilli*, as normal vaginal flora, have a central role in controlling body environment and preventing the growth of pathogens. Sucrose, by promoting the growth of *Lactobacilli*, accelerates the suppression of pathogenic bacteria. The aim of this research was to compare the effects of sucrose gel with those of metronidazole gel in treating women with bacterial vaginosis (BV).

**Methods:**

This triple-blind clinical trial (IRCT2016112631105N1) was conducted with 70 sexually active, premenopausal women diagnosed with bacterial vaginosis through meeting at least three out of four Amsel criteria. The subjects were randomly divided into two groups of 35 patients, one group treated with sucrose vaginal gel, and the other with metronidazole vaginal gel. The treatment period was 14 days for each group. At the end of the treatment period, the status of each woman’s improvement was determined by elimination at least three out of four Amsel criteria (homogeneous vaginal discharge, presence of clue cells > 20%, positive whiff test and vaginal pH value > 4.5), and clinical complaints and reported side effects of medication were recorded for the patients. Data were analyzed using the *t* test, chi-squared test and McNemar’s test).

**Results:**

The sucrose vaginal gel and metronidazole vaginal gel were not significantly different in reducing patients’ clinical complaints or in elimination at least three out of four of the Amsel criteria that were positive before treatment. With an 85.7% improvement rate with sucrose gel and an 88.5% improvement rate with metronidazole gel, the differences in therapeutic response were not significant, and neither was statistically different in improving the disease (*p* = 0.389).

**Conclusion:**

It seems that sucrose vaginal gel might be considered a possible alternative to metronidazole vaginal gel in the treatment of bacterial vaginosis.

**Trial registration:**

Iranian Registry of Clinical Trials, IRCT2016112631105N1. Registered on 27 December 2016.

## Background

Bacterial vaginosis (BV) is the most common vaginal disorder in women, and a syndrome associated with changes in vaginal ecology. In this clinical syndrome, *Lactobacillus* replacement occurs as normal vaginal flora with anaerobic bacteria and *Gardnerella vaginalis* [1, 2]. Vaginosis prevalence has been reported to be 20–49% in Africa, 11% in the UK and 15–30% in the USA [3]. Overall, the prevalence rate varies between 22% and 50% in various studies [4, 5].

Anaerobic bacteria are found in less than 1% of vaginal flora of healthy women. However, the anaerobic concentration in patients with BV reaches 100%. What triggers changes in normal vaginal flora has not been identified so far, but it is assumed that frequent vaginal alkalinization via factors such as vaginal douching and frequent sexual activity are effective in this area [6].

Four features comprise the Amsel criteria [7, 8], the principle of clinical diagnosis of the disease:

- gray, homogeneous, diluted vaginal discharge
- a positive whiff test (amine odor after treatment with potassium hydroxide)
- presence of clue cells in the vaginal fluid (> 20%)
- vaginal pH greater than 4.5

If untreated, this infection has adverse consequences, including spontaneous abortion, pre-term delivery, postpartum endometritis, the risk of sexually transmitted infections, pelvic inflammatory disease, postoperative infections and urinary tract infections [9–11]. The standard treatment for BV is oral and vaginal forms of metronidazole. However, beneficial effects of this drug should be weighed against its complications. Some side effects of these include nausea, abdominal pain and metallic taste in the mouth [12].

Because resistance to antibiotics is considered one of the greatest threats to public health, alternative therapies to antibiotics are essential for BV [13]. Currently, the use of herbal medicines and complementary medicine have particular importance in developing countries, and abundant research conducted by the World Health Organization in this field has led to the establishment of a strong scientific basis in this area [14]. Recently, findings of some studies have shown the antibacterial effect of sucrose in the vagina [13, 15]. *Lactobacilli*, as normal vaginal flora, have a central role in controlling the body’s ecosystem and preventing the growth of pathogens [16]. Normal flora makes use of sucrose as the main source of nutrition to produce lactic acid and hydrogen peroxide that cause the reduction in pH, resulting in an undesirable environment for the growth of pathogens. In addition, the osmolarity of sucrose causes the absorption of water and subsequently the disappearance of pathogenic bacteria [17]. As is warranted by the complications associated with chemical drugs [18], considering widespread use of traditional medications [19], the need for alternative treatment with fewer side effects, and the high prevalence of this infection among women, this study was conducted to compare the effects of sucrose gel with metronidazole gel in the treatment of women with bacterial vaginosis.

## Methods

This triple-blind, parallel randomized clinical trial (IRCT2016112631105N1) was conducted in 70 married women aged between 15 and 45 years, with bacterial vaginosis, who were patients at the women’s clinic at Ali ibn Abi Talib Hospital from May 2012 to March 2013. The facility is the largest medical center affiliated with the university in Zahedan, Iran. The sample size required to meet 80% power at 5% risk of type I error was 30 women per group, so a sample size of 35 per group was planned to account for a 10% loss to follow-up rate. This sample size was estimated considering 80% response to treatment (clinical symptoms) than the base line using this formula:

$$ n={\frac{\ \left[ z\alpha \sqrt{2{\pi}_1\left(1-{\pi}_1\right)}+ z\beta \sqrt{\pi_1\left(1-{\pi}_1\right)+{\pi}_2\left(1-{\pi}_2\ \right)}\right]}{\pi_1-{\pi}_2}}^2. $$

Therefore, 70 patients who met the inclusion criteria and had BV confirmed by a gynecologist who was not member of research team, were enrolled in the study. The study design and objectives were explained to the patients. All patients were asked to give written informed consent. Randomization was achieved using sealed, opaque, sequentially numbered envelopes developed from a random number generator. A midwife who was not involved in the recruitment of participants prepared the envelopes. As such, 35 patients were included in the group receiving sucrose gel and 35 patients composed the group receiving metronidazole gel based on a 1:1 ratio and in a single block.

### Inclusion criteria

The inclusion criteria were as follows: being sexually active, not being pregnant or breastfeeding at the time, not taking immunosuppressive drugs, not using an intrauterine device (IUD), not using vaginal douching and antibiotic therapy within 2 weeks prior to sampling, lack of any specific illness requiring treatment and the diagnosis of BV based on the presence of at least three out of four of the Amsel criteria (homogeneous vaginal discharge, presence of clue cells > 20%, amine odor when potassium hydroxide solution is added to the vaginal secretion, vaginal pH value > 4.5), and the absence of flagellated parasites of trichomonas or candida infection in the vaginal specimen based on laboratory tests. Exclusion criteria were as follows: not using the drug as prescribed, obligation to use antibiotics, or reluctance to continue.

Data were collected through questionnaires on demographics, a self-reporting sheet on patients’ symptoms, an observation checklist used during the first and second visits, observation using a microscope (Olympus, Japan) and pH test strips (Merck, Germany). The checklist included questions related to patient complaints and the Amsel criteria. Content validity was used for the observation checklist and the degree of agreement coefficient by kappa statistic was applied to assess its reliability; the agreement coefficient was 0.9. The Olympus microscope used is known to meet validity standards. Its validity was evaluated through calibrating the microscope.

To assess the reliability of the pH test strips, five standard specimens were prepared and the pH was measured for them. Validity was confirmed by correlation between standard levels and pH test strip results.

The study methodology was as follows:

- After history-taking, vaginal specimens were obtained from subjects in the lithotomy position; a sterile speculum without lubricant material was used.
- Each woman’s vagina and cervix were examined for evidence of inflammation and abnormal findings, and the vaginal discharge was assessed in terms of color, texture and smell.
- A discharge specimen from the upper part of the lateral wall of the vagina was placed on two slides using a swab. One to two drops of normal saline were added to the first specimen, which was examined under a microscope for the presence of clue cells and *Trichomonas vaginalis*.
- The second specimen was mixed with one drop of potassium hydroxide (KOH) 10% solution and examined for *Candida hyphae* and amine odor (specimens with flagellated parasites of *Trichomonas* or *Candida* infection were excluded from the study.)
- The pH of the vaginal discharge was determined using pH test strips.
- Patient complaints were recorded on the first-visit checklist.

Discharge specimen homogeneity was conducted by two midwifery experts (after training on specific procedures for this study), and their vaginal harvesting and microscopic observation techniques were verified by a laboratory sciences expert. Moreover, the research team gynecologist confirmed all diagnoses.

After definitive diagnosis of BV in the specimens, patients were randomly assigned by random numbers to the sucrose gel treatment group or the metronidazole gel treatment group. In this study, metronidazole gel 0.75% and sucrose gel 9% were prepared at the Laboratory of the Pharmacy School of Shahid Beheshti University in Tehran, Iran. The gels were inserted into identical 70-g tubes. Then, each tube was coded separately as A or B. In addition, the gels had no discernible differences in appearance, shape, color, or odor and were placed inside the completely identical tubes. Examiners, patients and the analysis team were unaware of which type of gel was within the tubes.

Study participants were advised to use the gel with an applicator morning and night for five days. A self-report sheet was given to patients to confirm that they had self-administered the treatment correctly each time; patients were supposed to bring the sheet to clinic visits. Participating women were advised to refrain from the following during the study:

- Intercourse without condoms
- Vaginal douching, spermicides or other vaginal medications
- Taking antibiotics other than what they may be using for the study

Patients were asked to refer 14 days after starting treatment. Amsel clinical criteria and patient complaints were re-evaluated, and results were recorded on the observation record forms. The absence of at least three out of four Amsel criteria 14 days after terminating treatment were indicative of treatment improvement [11, 20, 21]; any other result was considered as treatment failure. Participants, the gynecologist (assessor) and the statistician were blinded to group assignments from the beginning to the end of the study and data analysis. Data were analyzed using descriptive statistics (mean and standard deviation) and inferential statistics (*t* test, chi-squared test and McNemar’s test) using SPSS 21 software.

## Results

**Fig. 1:**
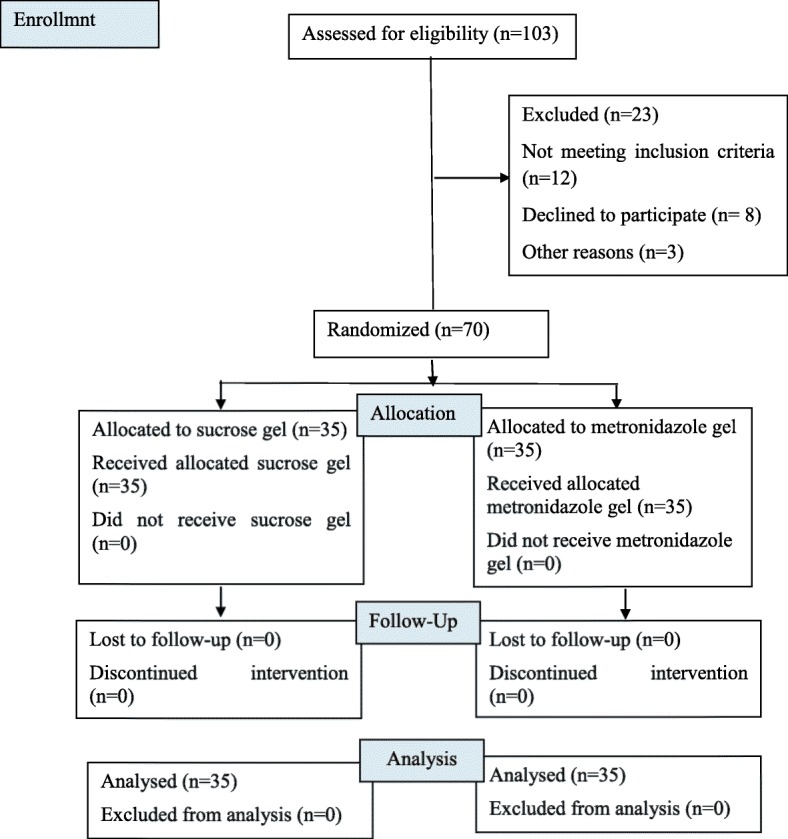
Consolidated Standards of Reporting Trials **(**CONSORT) flow diagram of the study

As mentioned, in this study, 70 women with BV were assigned to one of two groups of 35 patients. Figure 1 shows the Consolidated Standards of Reporting Trials (CONSORT) flow diagram of the study participants.

One group used sucrose vaginal gel and the other used metronidazole vaginal gel. The results showed no significant difference between the two groups in age, weight, age at onset of sexual activity and the number of pregnancies in both groups (Table 1).

**Table 1:** Comparison of mean and standard deviation of demographic characteristics and fertility status in two groups with bacterial vaginosis

Variable	Group treatment		(*P* values)*
	Sucrose gel Mean ± SD	Metronidazole Gel Mean ± SD	
Age (years)	32.31 ± 5.79	31.77 ± 5.07	0.678
Weight (kg)	64.17 ± 9.76	65.02 ± 9.90	0.716
Duration of marriage (years)	12.54 ± 7.42	10.32 ± 6.32	0.746
Age at first pregnancy (years)	19.62 ± 6.89	20.54 ± 6.04	0.557
The number of pregnancies	3.68 ± 2.45	3.22 ± 2.00	0.397

*Derived from *t* test

The maximum educational level was high-school completion in both groups with a frequency of 34.3% and 40% (*p* > 0.05), respectively, in the sucrose gel and metronidazole gel groups. In terms of occupation, 77.1% of subjects in the sucrose group and 88.6% of subjects in the metronidazole group were housewives (*p* > 0.05). The chi-squared test showed no significant difference between the two treatment groups in terms of education level and occupation (*p* > 0.05). Also, based on the chi-squared test, there was no statistically significant difference between the two treatment groups in terms of clinical complaints before and after treatment (*p* > 0.05). The McNemar test indicated a statistically significant difference between clinical complaints before and after treatment with sucrose gel and before and after treatment with metronidazole gel (Table 2).

**Table 2:** Comparison of absolute and relative frequency distribution of topics of patients’ clinical complaints before and after treatment

Criterion	A				Intra-group comparison before and after treatment (*P* values)*	B				Intra-group comparison before and after treatment (*P* values)*
	Before treatment		After treatment			Before treatment		After treatment		
	*N*	%	*N*	%		*N*	%	*N*	%	
Abundant vaginal discharge	35	100	8	22.9	*P* < 0.001	35	100	10	28.6	*P* < 0.001
Malodor	35	100	4	11.4	*P* < 0.001	35	100	2	5.7	*P* < 0.001
Itching	14	40	3	8.6	*P* = 0.001	13	37.1	5	14.3	*P* = 0.057
Dyspareunia	12	34.3	4	11.4	*P* = 0.021	9	25.7	1	2.9	*P* = 0.008
Abdominal pain	11	31.4	3	8.6	*P* = 0.008	17	48.6	5	14.3	*P* = 0.004
Group (*n*)	35		35			35		35		
Comparison of the two groups after treatment *P* > 0.05**										

*A* sucrose gel, *B* metronidazole gel, *N %* number and percentage of patients within each endpoint for each treatment group, *Group (n)* the number of patients in each group

*Derived from the McNemar test

**Derived from the chi-square test

Three of the four Amsel criteria - homogenous gray discharge, whiff test and pH > 4.5 - were reported 100% positive in both groups before treatment, and the chi-squared test showed no significant difference between the two groups (*p* > 0.05). The results comparison demonstrated that both treatments were effective in eliminating at least three out of four Amsel criteria, and no statistically significant difference was found between the two groups in terms of response to treatment (Table 3).

**Table 3:** Comparison of absolute and relative frequency distribution of the Amsel criteria before and after treatment

Criterion	A				Intra-group comparison before and after treatment (*P* values)*	B				Intra-group comparison before and after treatment (*P* values)*
	Before treatment		After treatment			Before treatment		After treatment		
	*N*	%	*N*	%		*N*	%	*N*	%	
Presence of vaginal discharge	35	100	8	22.9	*P* = 0.001	35	100	10	28.6	*P* < 0.001
Positive whiff test	35	100	13	37.1	*P* < 0.001	35	100	12	34.3	*P* < 0.001
Presence of clue cells	20	57.1	6	17.1	*P* = 0.001	22	62.9	13	37.1	*P* = 0.004
Vaginal pH > 4.5	35	100	10	28.6	*P* < 0.001	35	100	11	31.4	*P* < 0.001
Group (*n*)	35		35			35		35		
Comparison of the two groups after treatment *P* > 0.05**										

*A* sucrose gel, *B* metronidazole gel, *N %* number and percentage of patients within each endpoint for each treatment group, *Group (n)* the number of patients in each group

*Derived from the McNemar test

**Derived from the chi-square test

Treatment improvement was defined as the absence of at least three out of four Amsel criteria 14 days after terminating treatment. In this study, 85.7% of those undergoing sucrose therapy and 88.5% of those undergoing metronidazole therapy improved with BV treatment. The chi-squared test showed no significant differences in therapeutic response, and the two drugs were not significantly different in meeting criteria for treatment success (*p* > 0.05). Adverse effects related to drugs in both treatment methods were investigated: there were three cases (8.9%) of vaginal dryness and one case (2.9%) of itching in the metronidazole gel group and one case (2.9%) of vaginal dryness in the sucrose gel group.

## Discussion

The aim of this parallel, randomized, clinical trial was to compare the effect of sucrose gel and metronidazole gel in the treatment of clinical symptoms of bacterial vaginosis. The overall results showed that sucrose gel could be an alternative to metronidazole gel in the treatment of bacterial vaginosis. It has been documented that sucrose gel, with its restoration of normal vaginal flora, promotion of growth of *Lactobacilli* and suppression of pathogenic bacteria in patients with bacterial vaginosis, is considered a new choice for non-antibiotic-based treatment for BV [14, 16]. *Lactobacilli* are dominant, normal vaginal flora with the ability to inhibit adhesion strength and growth of pathogens, diminish pathogens’ access to nutrients and modulate the host immune response [22].

Marino et al., in a study carried out between 2002 and 2004, concluded that the use of *Lactobacillus* tablets can be effective in restoring normal vaginal flora. In patients with recurrent infections and those who frequently use antibiotics, *Lactobacilli* are reduced. Prescribing *Lactobacillus* was more effective than metronidazole [16].

There are limited studies in this regard; however, these studs have shown the positive effect of sucrose vaginal gel in improving symptoms of the infection. Xing et al., in a study of eight hospitals in China in 2008, found that metronidazole and sucrose are effective in the treatment of BV. Additionally, this study found that using sucrose vaginal gel could improve both the clinical and laboratory index of BV [23]. A randomized, double-blind, multi-center, parallel-group, phase III clinical trial conducted by Xiao et al. in 2015 found that the cure rate using sucrose gel was 80% while the cure rate using metronidazole was 70% [24]. In our study the improvement rate using sucrose gel and metronidazole gel were 85% and 88%, respectively, which indicates a greater improvement rate than the trial conducted by Xiao et al. in 2015.

Sucrose gel has no antibiotic properties - hence, there is no possibility of resistance - and it is a type of nutrition for *Lactobacilli* and was also shown to help the shifting of vaginal flora from the type in BV to *Lactobacilli* in an animal model [15]. Also, because the presence of *Lactobacilli* is an important factor in the prevention of infection, it is more effective than antibiotics such as metronidazole. For example, metronidazole not only prevents the growth of pathogenic bacteria, but also it simultaneously inhibits growth of *Lactobacilli*. This could be one of the causes of poor response to treatment and recurrence of infection [13].

The animal model of Hui et al., which showed the similarity of rhesus macaque vaginal microbial flora to that of patients with bacterial vaginosis, concluded that sucrose gel could cause changes in vaginal bacterial flora by decreasing the vaginal pH. In addition, they reported a significant increase in the relative frequency of *Lactobacillus* DNA, from 50.84% to 96.98% (*p* < 0.001). However, there was no change in the relative frequency of *Lactobacillus* DNA in the control group. They confirmed the important role of *Lactobacilli* in vaginal flora and thus in women’s health through the properties of probiotics, which prevent the growth of pathogens such as anaerobic bacteria, fungi and viruses by eliminating competition or producing organic acids and hydrogen peroxide. They introduced sucrose as a facilitator for growth of *Lactobacilli* [15].

Sucrose is a disaccharide composed of glucose and fructose carbohydrates [25]. Studies have reported an elevated survival rate of *Lactobacilli* in acidic conditions caused by glucose [26]. *Lactobacilli*, through glucose breakdown and hydrogen-ion production, are able to reduce pH, creating an unsuitable environment for pathogen growth [27, 28]. Monosaccharides, especially fructose, have a significant inhibitory effect on adhesion of pathogens to the mucosa [29]. In addition, the combination of fructose and glucose produces a significant increase in osmolarity and water reabsorption which, in turn, provides an unsuitable environment for fungi and other pathogens [30, 31].

Fructose and glucose also make up the main composition of honey; in fact, they comprise 85% to 95% of the sugar in honey [32]. The antimicrobial properties of honey, known for thousands of years, have been attributed to hydrogen peroxide, elevated osmolarity due to high levels of sugar like fructose, glucose, and sucrose and the acidity of its ingredients [33]. For example, the contents of honey, especially fructose, accelerate epithelial growth in wounds. It also eliminates the bad odor of wounds that is caused by lactic acid byproducts. In addition, the glucose oxidase activity in honey leads to low production of hydrogen peroxide which, in turn, prevents the growth of bacteria [34].

Given the aforementioned facts, it can be stated that the structure of sucrose has an effective role in inhibiting pathogen growth. Although the results of the present study and other limited research reveal the benefits of sucrose in the improvement of bacterial vaginosis, further studies are needed to determine the mechanism of action *Lactobacilli* in the presence of sucrose. Importantly, any such study should have a larger sample size to enhance the accuracy of the assessment. In addition, it is necessary to examine time dimensions in terms of infection recurrence levels to adequately determine the value of replacing routine antibiotics.

## Limitations

A limitation of this study was that two things were beyond the control of the researchers: subjects may have provided incorrect answers on the self-report sheet that assessed whether they used the medication correctly, and participants may not have complied with the study’s specialized health advice. Although being sexually active was one of our inclusion criteria, collecting data on this variable quantitatively might have provided more valid data on this risk factor. This limitation should be considered in future research.

### Limitation of findings in clinical practice

As sucrose gel is not readily available in many pharmacologic environments, it may be more costly and more difficult for women to obtain it than to obtain a prescription for metronidazole gel.

## Conclusion

The findings gleaned from the present study demonstrated that sucrose vaginal gel is effective in treating bacterial vaginosis. According to the positive effects of sucrose and its compounds on normal vaginal flora, it seems that further studies can contribute to achieving beneficial outcomes in the treatment of infections in women, especially bacterial vaginosis.

## Abbreviations

BV: Bacterial vaginosis
CONSORT: Consolidated Standards of Reporting Trials
IRCT: Iranian Registry of Clinical Trials
KOH: Potassium hydroxide
